# Bacterial upper respiratory tract infections in Brazil: bacterial resistance, human resistance, scientific darkness

**DOI:** 10.1016/j.bjorl.2021.01.001

**Published:** 2021-01-14

**Authors:** Otavio Bejzman Piltcher, José Faibes Lubianca Neto

**Affiliations:** a Universidade Federal do Rio Grande do Sul (UFRGS), Faculdade de Medicina (FAMED), Disciplina de Oftalmologia e Otorrinolaringologia, Porto Alegre, RS, Brazil; b Universidade Federal de Ciências da Saúde de Porto Alegre, Disciplina de Otorrinolaringologia, Porto Alegre, RS, Brazil; c Universidade Federal de Ciências da Saúde de Porto Alegre, Pós-Graduação em Pediatria, Porto Alegre, RS, Brazil; d Santa Casa de Misericórdia de Porto Alegre, Serviço de Otorrinolaringologia, Porto Alegre, RS, Brazil; e Hospital da Criança Santo Antônio, Serviço de Otorrinolaringologia Pediátrica, Porto Alegre, RS, Brazil

Antibiotic therapy options for bacterial upper respiratory tract infections (URTIs) such as pharyngotonsillitis, rhinosinusitis and acute otitis media, are based on the results of sensitivity tests from large surveillance studies. However, these studies evaluate microorganisms from invasive infections (for instance, SIREVA - System of Surveillance Networks for Agents Responsible for Pneumonia and Bacterial Meningitis in Latin America and the Caribbean). There is a lack of studies seeking systematically for the type of bacteria and its degree of resistance among upper airways (UA) infections. This is true specially in Brazil. There are obvious ethical concerns regarding this type of study, forcing us to keep basing antimicrobial treatments on extrapolations of bacterial susceptibility associated with other infections or on international microbiological data that may not reflect our reality. Not even indirect measures to determine our flora are encouraged, such as the rhinopharynx swab culture, of which isolates have good correlation with the bacteria that cause AOM and acute rhinosinusitis, for example.^1^, ^2^, ^3^

Meanwhile, we worry daily about the increase in bacterial resistance of the main UA microorganisms for macrolides, penicillin and fluoroquinolones. This observation results in suggestions and pressure for changes in the usual treatments protocols, such as changing the antimicrobial and/or increasing doses. However, for decades we have been surprised with the fact that many of our day-to-day patients continue to show good clinical evolution while receiving the same antibiotics and at the same recommended standard doses. How can we explain this phenomenon? Are bacterial strains of *S. pneumoniae* that cause invasive systemic infections different from those associated with UA infections, both in virulence and resistance profile? Would the local immune system (respiratory nasal epithelium and middle ear epithelium) be different from that of other parts of the body, leading to spontaneous resolution regardless of whether the bacteria is resistant to antibiotics or not? Would the microbiological efficacy be dissociated from clinical efficacy, as when it is observed that more than 60% of patients with AOM show clinical success despite having positive cultures in tympanocentesis performed between the 3rd and 7th days of treatment? Would it be acceptable to keep extrapolating the resistance for a full family of antimicrobials according to the response for an isolated representative agent when there is evidence of significant differences between different elements of a same group of drugs for example pneumococcal resistance to penicillin vs resistance to amoxicillin^4^

Finally, shouldn’t we be questioning whether such favorable rates of resolution with the usual antimicrobials at standard regimens do not represent the unacceptable percentage of misdiagnosed viral URTI treated with antibiotics?^5^, ^6^, ^7^

Clearly as doctors, in the field of URTIs, we are sailing in the dark, without a GPS or Waze. In an incomprehensible way and luckily for everyone, there seems to be an invisible path leading to light, that is, clinical resolution. It is imperative, even for us to respect more the threat of generating a resistant microorganism, to know how sensitive the bacteria that cause our otitis, acute rhinosinusitis and pharyngotonsillitis are, similarly, for instance, to the Israeli team consisting of Leibovitz, Dagan R and colleagues in their series of studies in AOM.^8^, ^9^ If not with the ethically contested “double tap”, at least with a tympanic paracentesis at the time of diagnosis, or with swabs of the middle meatus or palatine tonsils before treatment. These studies are not only essential now, but also in the future, from time to time, to analyze and recognize changes in the resistance profile of our bacteria.

Being as clear as possible, no one is minimizing bacterial resistance in the upper airways or advocating laxity in this matter. We are in fact pointing to the importance of the topic and for the urgency in advocating and running further local studies that will define which bacteria we are dealing with and what are our truly effective weapons to manage these infections.

In 2020, despite so many scientific advances in medicine, the only unequivocal assertion is that the greatest resistance of all is that of health professionals and the population in general to assimilate the need for urgent changes in the understanding and management of these infections.

## Conflicts of interest

The authors declare no conflicts of interest.
